# Research hotspots and trends of mesenchymal stem cell-derived extracellular vesicles for drug delivery: a bibliometric and visualization analysis from 2013 to 2023

**DOI:** 10.3389/fcell.2024.1412363

**Published:** 2024-10-30

**Authors:** Tianyuan Zhao, Yuhao Mu, Haobin Deng, Kaini Liang, Fanfan Zhou, Qiyuan Lin, Fuyang Cao, Feifei Zhou, Zhen Yang

**Affiliations:** ^1^ Department of Orthopaedics, Peking University Third Hospital, Beijing, China; ^2^ School of Medicine, Nankai University, Tianjin, China; ^3^ Department of Oncology, Liuzhou People’s Hospital Affiliated to Guangxi Medical University, Liuzhou, China; ^4^ School of Biomedical Engineering, Tsinghua University, Beijing, China; ^5^ Arthritis Clinical and Research Center, Peking University People’s Hospital, Beijing, China; ^6^ Shanxi Key Laboratory of Bone and Soft Tissue Injury Repair, Second Hospital of Shanxi Medical University, Taiyuan, Shanxi, China

**Keywords:** research hotspots and trends, mesenchymal stem cell, extracellular vesicles, bibliometric, visualization

## Abstract

**Introduction:**

Our study aims to provide a comprehensive overview of mesenchymal stem cell-derived extracellular vesicles (MSC-EVs) in drug delivery research, focusing on the period between 2013 and 2023. Given the increasing global interest in this field, we utilized bibliometric tools to explore publication trends, key contributors, and thematic research clusters.

**Methods:**

Data was collected from the Web of Science (WoS) database, and an in-depth bibliometric analysis was conducted using VOSviewer. The analysis encompassed bibliographic coupling, co-citation, co-authorship, and co-occurrence trends, offering a structured insight into global research activity. We also employed Citespace to further analyze thematic clusters in this domain.

**Results:**

Our analysis revealed a total of 1,045 publications related to MSC-EVs in drug delivery over the past decade, showing a steady increase in research output. China led in publication count, H-index, prolific authors, and research funding, while the United States ranked highest in total citations, average citation counts, and H-index performance. Pharmaceutics emerged as the leading journal by publication volume, with the Journal of Controlled Release having the strongest total link strength. Top institutions driving research included Shanghai Jiao Tong University, Zhejiang University, and Harvard University. VOSviewer analysis identified four major research clusters: tissue engineering, cancer, neurological diseases, and targeted delivery. Citespace analysis refined this further into ten thematic areas, including differentiation, tissue regeneration, and drug resistance.

**Discussion:**

This bibliometric assessment provides a holistic visualization of the research landscape for MSC-EVs in drug delivery, underlining the significant contributions of China and the United States. Our findings underscore the increasing global importance of MSC-EV research and highlight emerging themes that will likely guide future research directions. The insights from this study offer a foundational framework for identifying nascent frontiers in MSC-EV-based drug delivery.

## 1 Introduction

Mesenchymal stem cells (MSCs) are multipotent stromal cells that can differentiate into a variety of cell types, including osteoblasts (bone cells), chondrocytes (cartilage cells), adipocytes (fat cells), and myocytes (muscle cells) (Ala, 2023). They are found in various tissues such as bone marrow, adipose tissue, umbilical cord blood, and dental pulp (Wu et al., 2023; Shen et al., 2019; Ma et al., 2024; Kim et al., 2013). MSCs have gained significant attention in regenerative medicine and cell-based therapies due to their immunomodulatory properties, anti-inflammatory effects, and ability to promote tissue repair and regeneration (Porada and Almeida-Porada, 2010; Barry and Murphy, 2013). While MSCs hold considerable promise for tissue regeneration and repair applications, several limitations hinder their therapeutic efficacy. These challenges include poor MSC homing and engraftment at target sites, limited cell survival and retention post-transplantation, immune rejection, variability in potency and phenotype among donor sources, and concerns regarding long-term safety, such as tumorigenesis or ectopic tissue formation (Mansoor et al., 2019). Additionally, the complex interactions between MSCs and the host microenvironment can influence their functional properties and therapeutic outcomes, further complicating their clinical translation.

Increasing evidence suggests that the therapeutic effects of MSCs are largely mediated through paracrine mechanisms, and extracellular vesicles (EVs) (including exosomes, microvesicles, microparticles and apoptotic bodies) have been shown to playing a significant role in this process (Théry et al., 2018). These small membrane-bound vesicles secreted by MSCs contain a diverse cargo of bioactive molecules such as proteins, lipids, and nucleic acids, which exert therapeutic effects on target cells and tissues (Wu et al., 2021). EVs are seen as an alternative approach to stem cell therapy, offering numerous advantages such as enhanced stability, decreased manufacturing expenses, and improved convenience in sterilization and storage (Yuan et al., 2022). EVs facilitate intercellular communication and modulate various physiological processes including inflammation, immune response, angiogenesis, and tissue repair (Tan et al., 2024).

Additionally, MSC-derived EVs (MSC-EVs) are widely utilized as drug carriers in treating various diseases, offering several advantages including enhanced stability, prolonged circulation time, and reduced immunogenicity (Lin et al., 2022). Researchers generally use two methods to load therapeutic agents into extracellular vesicles (EVs): the direct method, where therapeutic agents are inserted into purified exosomes, and the indirect method, where parental cells are genetically modified or co-cultured with a therapeutic agent to produce engineered exosomes. Various therapeutic components, such as drugs, proteins, and RNA, are loaded into MSC-derived EVs, enhancing targeting effects to selectively reach target cells or tissues with minimal systemic side effects. Moreover, their exquisite biocompatibility and ability to cross biological barriers make them attractive candidates for targeted drug delivery. Recently, MSC-EVs have been widely used in bone fracture (Behera et al., 2021), osteoarthritis (Liu et al., 2018), osteoporosis (Yahao and Xinjia, 2021), spinal cord injury (Nakazaki et al., 2021), sciatic nerve injury (Bucan et al., 2019), intervertebral disc degeneration (Hu et al., 2021), ischemic stroke (Zhang et al., 2021), traumatic brain injury (Moore et al., 2019), Alzheimer’s disease (Chen et al., 2021), Parkinson’s disease (Huang et al., 2022) and etc., Nevertheless, there is inadequate research examining the quantitative and qualitative attributes of global trends and focal points in MSC-EVs for drug delivery. Therefore, it is imperative to assess the present state and trajectories of MSC-EVs in drug delivery, as this can anticipate promising areas of interest and avenues for advancement in this domain.

As the cornerstone of scientific inquiry, publications serve as a crucial barometer of research trends and contributions. Leveraging the characteristics of literature metrology and comprehensive literature databases, bibliometric analysis offers both qualitative and quantitative insights into the trajectory of research endeavors over time. Through this analytical approach, one can forecast the evolution of a specific field by examining the contributions of authors, journals, institutes, countries, and regions. Moreover, bibliometric analysis plays a pivotal role in informing the formulation of clinical policies and the establishment of guidelines. Currently, this viable methodology has been effectively employed in appraising research trends within nanomedicine (Wang et al., 2024), depressive disease (Liu et al., 2024), tumor (Song et al., 2023), and osteoarthritis (Yang Z. et al., 2022) and so on. While we conducted a prior examination of global research trends in EVs derived from stem cells spanning from 1991 to 2021, and Zhang et al. provided a review of current research on MSC-EVs from 2009 to 2021, neither study comprehensively nor promptly delved into the realm of MSC-EVs within the field of drug delivery (Zhang X. et al., 2022). The objective of our study was to assess the contemporary status and worldwide trends pertaining to the utilization of MSC-EVs in drug delivery.

## 2 Materials and methods

### 2.1 Search strategy and data collection

All publications sourced from Web of Science (WoS), encompassing Science Citation Index Expanded (SCIE), Arts and Humanities Citation Index, Social Sciences Citation Index, Conference Proceedings Citation Index-Science, Emerging Sources Citation Index, Conference Proceedings Citation Index-Social Science and Humanities, Current Chemical Reactions, and Index Chemicus, constituting a repository of over 12,000 international academic journals, represent one of the most comprehensive and authoritative database platforms for accessing global academic information (Lin et al., 2022). These publications underwent bibliometric analysis in accordance with our prior studies (Yang Z. et al., 2022; Yang et al., 2023a; Yang et al., 2023b).

All published papers were gathered from WoS, with the database’s expiration date set to 9 February 2024. The search criteria included the following terms: “mesenchymal stem cell” OR “mesenchymal stem cells” AND “extracellular vesicles” OR “exosomes” OR “microvesicles” OR “microparticles” OR “ectosomes” OR “oncosomes” OR “apoptotic bodies” AND “drug delivery,” limited to publications in English and categorized as either articles or reviews. The search period was defined as 1 January 2013, to 31 December 2023. Additionally, the detailed information regarding specific countries or regions was refined by indexing them within the WoS. The inclusion criteria stipulated that manuscripts must focus on the theme of MSC-EVs for drug delivery, be written in English, and categorized as either articles or reviews. Exclusion criteria included themes unrelated to MSC-EVs for drug delivery and article types such as briefings, news, and meeting abstracts. For *in vivo* studies, the search strategy was further refined by adding the term “AND *in vivo*” to the above search criteria. This resulted in a total of 286 published papers specifically focusing on the *in vivo* applications of MSC-EVs for drug delivery. The same inclusion and exclusion criteria were applied to ensure that only relevant articles or reviews were included in this subset of the analysis.

All publication records, comprising publication year, title, authors’ names, affiliations, nationalities, abstracts, keywords, and journal names, were extracted from the WoS database and saved as.txt files. These files were subsequently imported into Excel 2021. Coauthors (TZ and YM) independently reviewed and extracted data from these publications. Any discrepancies were resolved through discussion with experts to achieve consensus. Finally, all authors independently cleaned and analyzed the data using GraphPad Prism 8.

### 2.2 Bibliometric analysis and visualization

The intrinsic functions of WoSCC were utilized to characterize the fundamental features of eligible studies. Specifically, the publication frequency and citation counts were analyzed and visualized. GraphPad Prism 8 and Origin 8 were employed for the bibliometric analysis. Initially, the year was plotted on the *x*-axis and the number of documents published each year was plotted on the *y*-axis to examine the trend of publication output over time. Relative research interest (RRI), defined as the number of publications in a specific field per year relative to all field literature, was calculated (Shah et al., 2022). Utilizing a combination of R software, including Python, NumPy, SciPy, and Matplotlib, a world map was generated to visualize publication trends. Then a trend chart depicting the annual publication volume of the top ten countries is generated by using Origin 8 software. Additionally, the H-index, which denotes a researcher who has published H papers and has been cited at least H times, was computed to assess the impact of scientific research (Hu et al., 2022).

In this study, we employed VOSviewer software from Leiden University in the Netherlands to comprehensively visualize bibliometric networks of the publications. We conducted detailed analyses of bibliographic coupling, co-citation, and co-occurrence, and presented the results using VOSviewer. The key parameter settings mainly focused on determining the minimum number of documents, citations, or occurrences. Specifically, for bibliographic coupling analysis, we set the minimum number of documents for Country, Journal, Author, and Institution at more than 2. For co-citation analysis, we defined the minimum number of citations for Author, Reference, and Journal as more than 10. Regarding co-authorship analysis, we established the minimum number of documents for Country, Author, and Institution to be more than 2. Additionally, for co-occurrence analysis, keywords were identified as words appearing more than 2 times in titles/abstracts across all papers.

Furthermore, CiteSpace (6.2.R6) was employed to construct a dual-map overlay for journals, visualize diagrams of country/regional collaboration, institutional collaboration, and author collaboration, conduct cluster analysis of co-cited keywords, and identify references and keywords with intense citation bursts. This software, developed by Professor Chen C., was configured with the following parameters: link retaining factor (LRF = 3), look back years (LBY = 5), e for top N (e = 1), time span (2013–2023), years per slice (1), links (strength: Cosine, scope: Within slices), selection criteria (g-index: k = 10), and minimum duration (MD = 2 for keywords; MD = 5 for references).

The bibliometric analysis in this study was mainly conducted using VOSviewer and CiteSpace software. No custom code was used in this process. The parameters and settings applied in both tools are available upon request. Raw data and generated outputs can also be provided for further validation and reproducibility purposes.

## 3 Results

### 3.1 Trend of global publications

**FIGURE 1:**
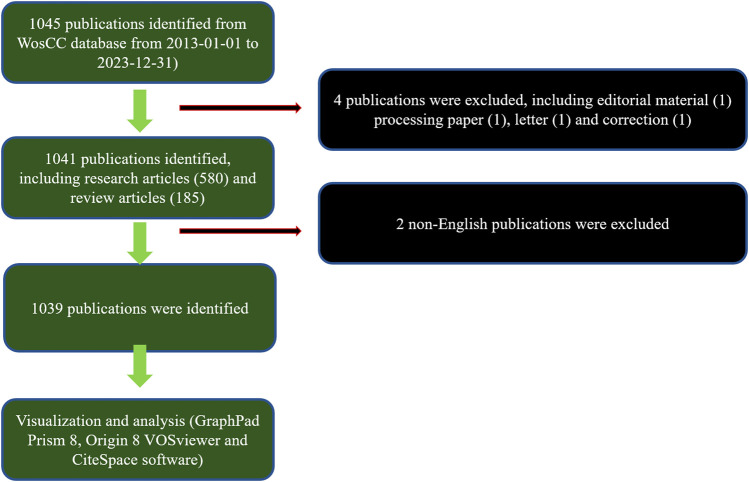
Flowchart depicting the process of publications selection.

**FIGURE 2:**
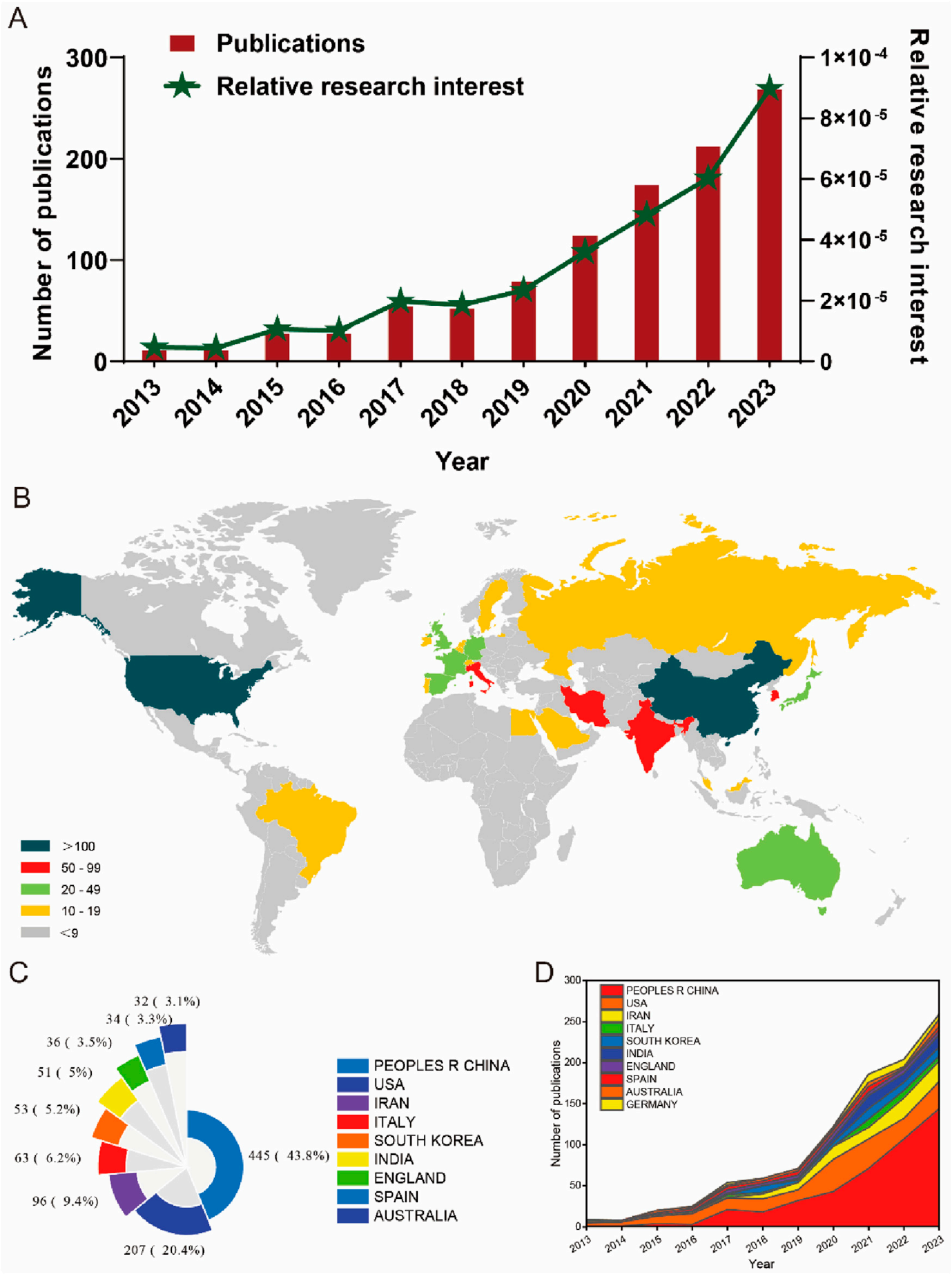
Global trends and the contributions of countries/regions to the research field of MSC-EVs for drug delivery from 2013 to 2023. **(A)** The yearly count of publications related to MSC-EVs for drug delivery from 2003 to 2023. **(B)** A world map illustrating the distribution of MSC-EVs for drug delivery from 2003 to 2023. The total **(C)** and yearly **(D)** number of publications in the top 10 most productive countries from 2013 to 2023 are analyzed.

In the realm of global scholarly output, a comprehensive review of literature from 2013 to 2023 yielded 1,045 publications aligning with the established search parameters. Subsequent refinement of this corpus involved the exclusion of editorial materials (1), processing papers (1), letters (1), and corrections (1), along with the removal of two non-English studies. This meticulous process resulted in the identification of 1,039 pertinent studies, as depicted in Figure 1. Analyzing the temporal progression from 2013 to 2023, a consistent upward trajectory in global publication volume is delineated. The annual count of publications experienced a notable increase, escalating from 11 in 2013 to 268 in 2023. Particularly in the last 5 years (2019–2023), this ascending trend in publication volume has intensified, as illustrated in Figure 2A. Furthermore, the analysis of contributions by countries and regions revealed a diverse international involvement, with 73 countries/regions contributing to the literature in this field, as shown in Figure 2B. Among these, the top 10 countries/regions displayed a significant predominance in publication output. China led with a remarkable contribution of 445 papers, accounting for 43.8% of total publications, followed by the United States (207, 20.4%), Iran (96, 9.4%), Italy (63, 6.2%), South Korea (53, 5.2%), and India (51, 5%), as detailed in Figures 2C, D.

### 3.2 Quality of publications of different countries/regions

**FIGURE 3:**
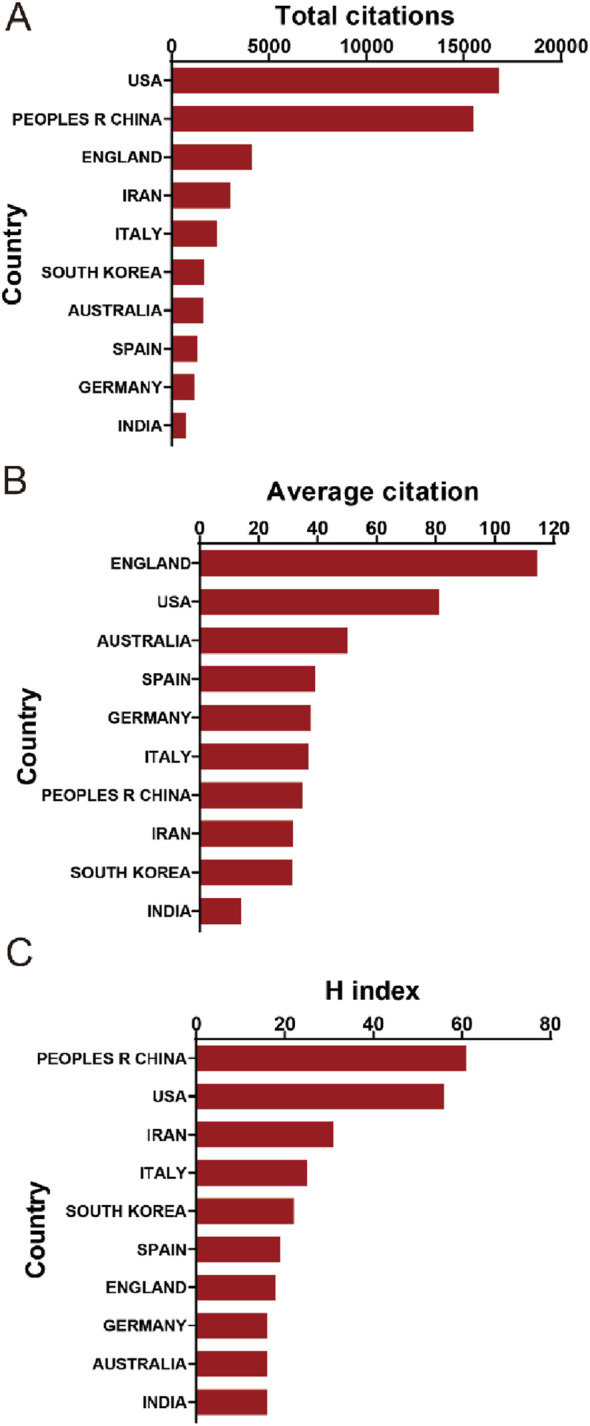
**(A)** The top 10 countries/regions in terms of total citations regarding MSC-EVs for drug delivery from 2013 to 2023. **(B)** The top 10 countries/regions in terms of the average citations per publication related to MSC-EVs for drug delivery from 2003 to 2023. **(C)** The top 10 countries/regions in terms of the publication H-index related to MSC-EVs for drug delivery from 2013 to 2023.

In the context of total citation frequency analysis, publications originating from the United States demonstrated the highest aggregate citation count, amassing a total of 16,800 citations. China occupied the second position with an impressive citation tally of 15,489, succeeded by England with 4,118 citations, Iran with 3,028, and Italy with 2,329, as delineated in Figure 3A. Moreover, when examining the average citation frequency, publications from England emerged as the frontrunner with an average citation frequency of 114.39. The United States followed closely, ranking second with an average citation frequency of 81.16. Australia (50.06), Spain (30.15), and Germany (37.61) also featured prominently in this metric, as highlighted in Figure 3B. Additionally, in terms of the H-index, a key indicator of research impact, publications from China led with the highest H-index of 61. The United States followed with an H-index of 56, Iran with 31, Italy with 25, and South Korea with 22, underscoring the significant research impact of these countries in the domain, as illustrated in Figure 3C.

### 3.3 Analysis of country/regional and institution collaboration

**FIGURE 4:**
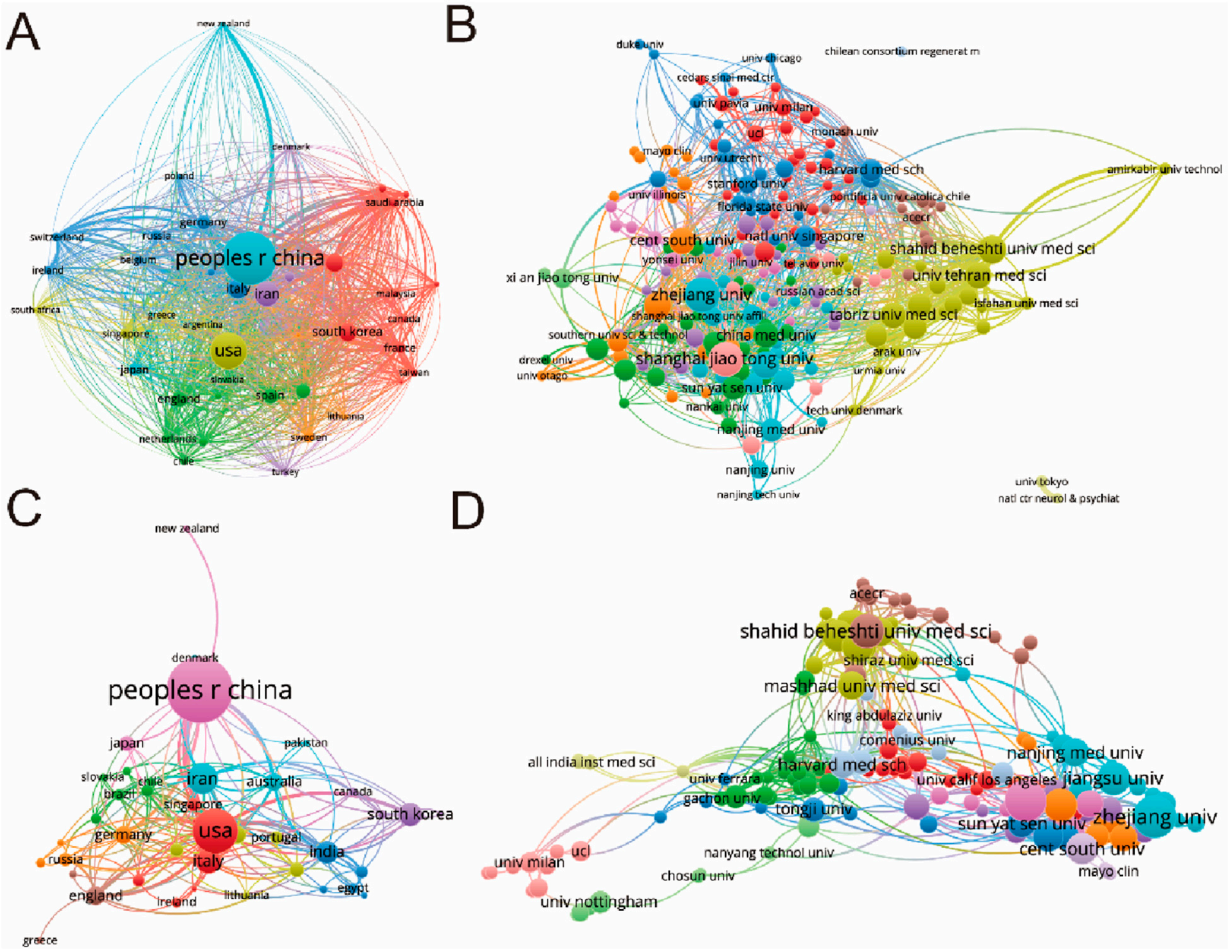
Mapping of countries/regions and institutions associated with MSC-EVs for drug delivery spanning from 2013 to 2023. **(A)** Examination of country/regional collaboration utilizing Vosviewer. **(B)** Analysis of institutional collaboration using Vosviewer. **(C)** Authorship-country collaboration analysis via Vosviewer. **(D)** Authorship-institution collaboration analysis via Vosviewer. Nodes represent countries/regions and institutions, varying in size based on the number of publications attributed to each. Connecting lines denote cooperation, with line thickness indicating collaboration strength; thicker lines denote closer cooperation.

In this comprehensive analysis, a global collaboration network was meticulously examined, focusing on publications with a minimum threshold of three documents per country. This scrutiny, conducted through VOS viewer and depicted in Figure 4A, involved publications from 42 countries. China emerged as a dominant force, showcasing the highest output area and robust international collaboration, as evidenced by its pronounced interactions with other nations. The five foremost countries, in terms of total link strength, were China (524,072 times), the United States (254,718 times), Iran (141,744 times), India (109,942 times), and South Korea (105,309 times).

Further analysis, delineated in Figure 4B, was directed towards academic institutions, with a focus on those with a minimum of three documents and a maximum of 25 organizations per document. This investigation encompassed 216 institutions, again employing VOS viewer for analytical insights. The institutions demonstrating the greatest total link strength were Zhejiang University (49,430 times), Shanghai Jiao Tong University (44,068 times), Sichuan University (43,342 times), Tehran University of Medical Sciences (39,876 times), and Jiangsu University (34,521 times).

Additionally, a comprehensive summary of funding sources for stem cell research in meniscal regeneration is presented in Table 1. The National Natural Science Foundation of China leads with support for 283 articles, followed by the United States Department of Health and Human Services (84 articles) and the National Institutes of Health (NIH), United States (83 articles). Notably, the top three funding sources corroborate the earlier findings, highlighting the significant contributions from both China and the United States in this research domain.

**TABLE 1:** The top 10 well-represented research areas.

Rank	Research areas	Records	Percentage (%)
1	Pharmacology Pharmacy	271	26.083
2	Materials Science	190	18.287
3	Science Technology Other Topics	174	16.747
4	Cell Biology	167	16.073
5	Chemistry	164	15.784
6	Research Experimental Medicine	130	12.512
7	Biochemistry Molecular Biology	113	10.876
8	Engineering	103	9.913
9	Oncology	89	8.566
10	Biotechnology Applied Microbiology	85	8.181

### 3.4 Co-authorship analysis of country, and institution

The co-authorship analysis, a critical component of understanding collaborative patterns in research, was conducted on a select group of countries and institutions. This analysis employed VOS viewer as the primary tool for visualization and assessment. Regarding the country-based co-authorship analysis, 32 countries, each contributing more than three papers, were selected for scrutiny. The results of this analysis are encapsulated in Figure 4C. The United States emerged as the leader in co-authorship strength, boasting a total link strength of 152 times. China followed with a total link strength of 93 times. Other notable countries included Iran (58 times), England (42 times), India (42 times), and Italy (also at 42 times), all demonstrating significant collaborative linkages in the research community. The institutional co-authorship analysis extended to 216 institutions, each with more than three documents to their credit. The findings, illustrated in Figure 4D, identified Shahid Beheshti University of Medical Sciences as having the highest total link strength at 49 times. Harvard Medical School followed closely with a strength of 46 times. Other institutions in the top five included Tehran University of Medical Sciences (44 times), Massachusetts General Hospital (42 times), and Shanghai Jiao Tong University (32 times). This comprehensive co-authorship analysis at both country and institutional levels underscores the interrelation and collaborative efforts in the field, highlighting key players and the strength of their scientific networks.

### 3.5 Analysis of authors collaboration

**FIGURE 5:**
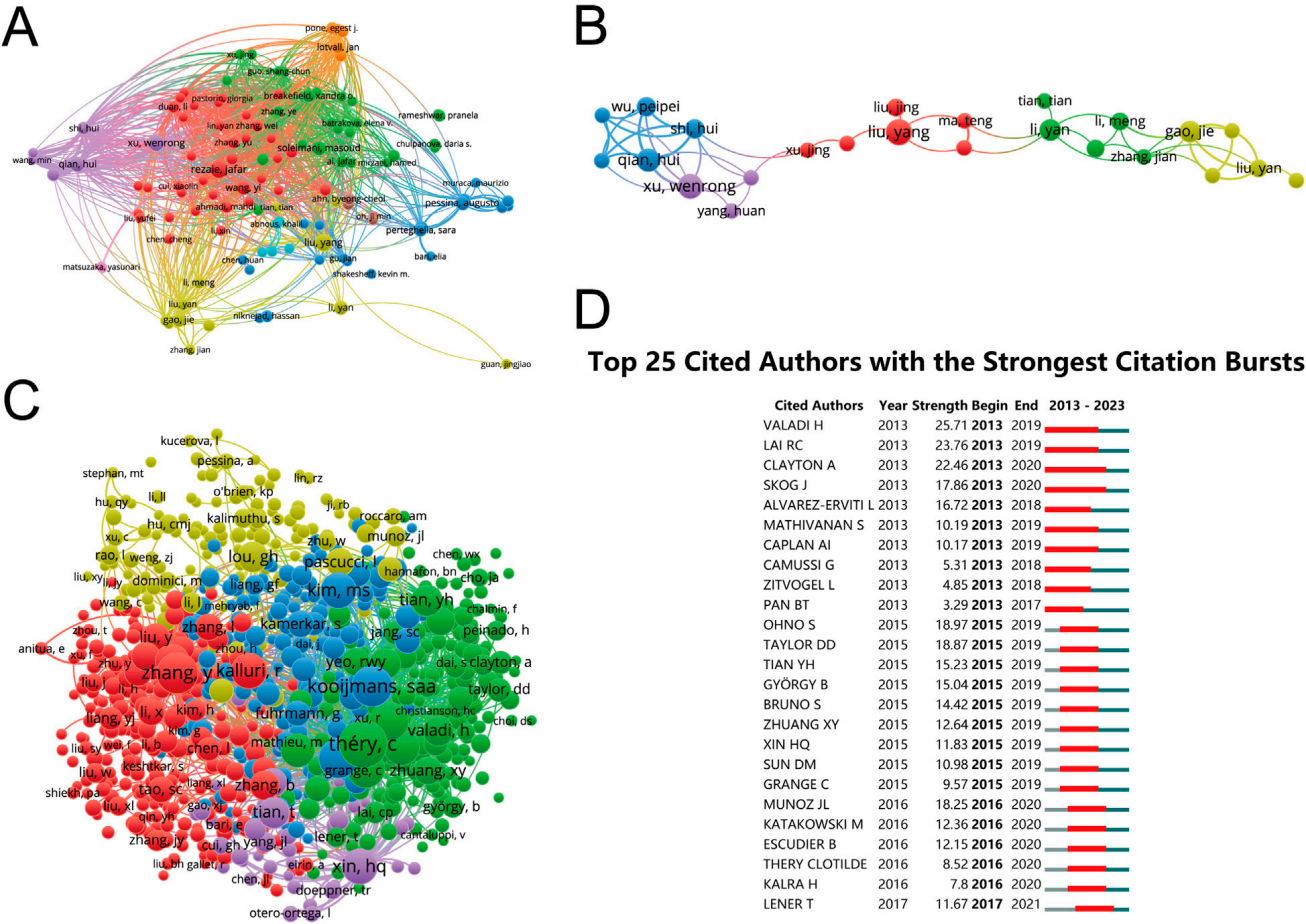
Network visualization of author collaboration analysis concerning MSC-EVs for drug delivery spanning from 2003 to 2023. **(A)** Author collaboration analysis conducted using Vosviewer. **(B)** Visualization diagram illustrating authorship-author analysis based on Vosviewer. **(C)** Network visualization diagram illustrating cocited-author analysis based on Vosviewer. **(D)** Identification of the top 25 cited authors exhibiting the most pronounced citation bursts in publications related to MSC-EVs for drug delivery. Author collaborations are depicted by nodes, with the size of each node scaling in accordance with the number of collaborations. Collaboration relationships are represented by connecting lines between nodes.

The investigation into authorship productivity, encompassing publications from 118 authors, revealed a remarkable level of output. Figure 5A illustrates these findings, with the most prolific authors being Qian, Hui (total link strength = 11,816 times), Shi, Hui (11,176 times), Xu, Wenrong (9,930 times), Liu, Yang (9,011 times), and Schiffelers, Raymond, M (8,835 times). This analysis highlights the individual contributions that have significantly shaped the research landscape in this field.

The co-authorship analysis, involving 118 authors with over 3 documents each, focused on the collaborative networks within the research community. As shown in Figure 5B, the top collaborators were identified based on their total link strength: Pessina, Augusto (20 times), Pascucci, Luisa (19 times), Qian, Hui (19 times), Shi, Hui (17 times), and Alessandri, Giulio (16 times). This analysis underscores the importance of collaborative endeavors in advancing research.

Co-citation analysis, which included 915 authors with a minimum of 20 documents, was conducted to understand the inter-relationships based on citation frequency. The findings in Figure 5C point to the most co-cited authors: Thery, C (total link strength = 23,454 times), Kooijmans, Saa (21,191 times), Haney, Mj (18,839 times), Alvarez-Erviti, I (16,181 times), and Kim, Ms (14,756 times). This aspect of the analysis sheds light on the influential works and authors within the field, reflecting their impact and relevance.

The citation burst analysis, summarized in Figure 5D, identified key publications that experienced a surge in citations over a specific period, indicating a spike in interest and relevance in the field. The study by Vakadi H exhibited the strongest citation burst (strength of 25.71) from 2013 to 2019, followed by Lai RC (23.76) and Clayton A (22.46). This analysis provides insights into the evolving trends and pivotal studies driving research focus over time.

### 3.6 Analysis of research areas and journals

**FIGURE 6:**
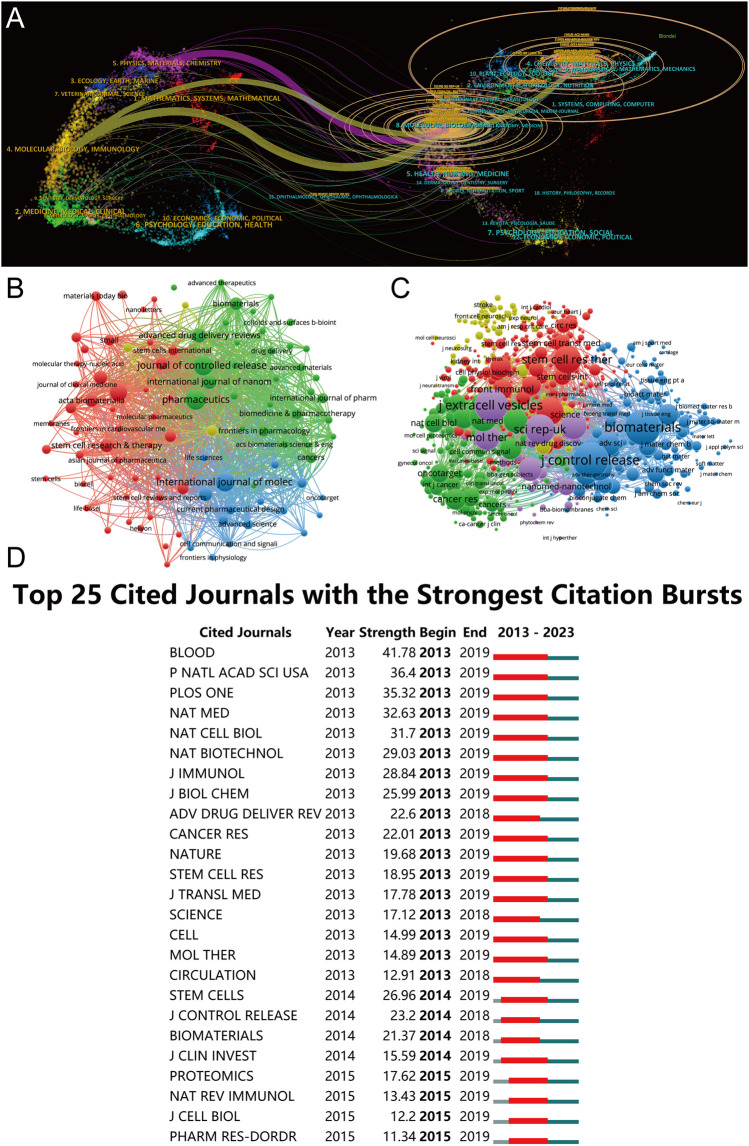
Articles published in various journals on MSC-EVs for drug delivery from 2013 to 2023. **(A)** Dual-map overlay depicting journals relevant to MSC-EVs for drug delivery. **(B)** Bibliographic analysis of journals utilizing VOSviewer. **(C)** Network map illustrating journals that were co-cited based on VOSviewer. **(D)** Identification of the top 25 cited journals exhibiting the most pronounced citation bursts in publications.

In the realm of academic publishing, the 10 most prolific journals contributing to this study have been systematically chronicled in Table 2. An intricate dual-map overlay, delineating the journals associated with MSC-EVs for drug delivery, has been meticulously constructed and is presented in Figures 6A, B. Pharmaceutics leads the cohort with a commendable tally of 37 publications, followed closely by the Journal of Controlled Release with 35 publications, International Journal of Molecular Sciences with 34, Frontiers in Bioengineering and Biotechnology with 27, and International Journal of Nanomedicine contributing 19 articles.

**TABLE 2:** The top 10 authors with the most publications on MSC-EVs for drug delivery from 2013 to 2023.

Rank	Highly published authors	Article counts	Percentage (%)
1	Zhang Y	18	1.732
2	Liu Y	16	1.54
3	Li Y	15	1.444
4	Wang J	15	1.444
5	Wang Y	15	1.444
6	Li X	11	1.059
7	Liu J	10	0.962
8	Rezaie J	10	0.962
9	Li M	9	0.866
10	Wang L	9	0.866

A thorough co-citation analysis of journals, employing VOSviewer and adhering to a criterion that excludes journals with fewer than 20 citations, yielded significant insights. As elucidated in Figure 6C, this analysis encapsulated 832 journals in total link strength. The forefront of this collection is dominated by five journals: the Journal of Controlled Release (3,986 citations), Biomaterials (2,934 citations), Journal of Extracellular Vesicles (2,536 citations), Scientific Reports (2,118 citations), and Stem Cell Research and Therapy (1,810 citations), each demonstrating substantial citation influence.

In the context of citation bursts over the past decade, CiteSpace analysis highlighted 25 journals, as depicted in Figure 6D. This analysis underscores the journals that garnered heightened scholarly attention within specific timeframes. Notably, the journal Blood exhibited the most pronounced citation burst, with an impressive strength of 41.78, spanning from 2013 to 2019.

Furthermore, a comprehensive enumeration of research orientations is collated in Table 3, utilizing VOSviewer for precision. The predominant research fields, as revealed, encompass pharmacology and pharmacy, materials science, science and technology in other topics, cell biology, and chemistry. These focal areas not only reflect the current research milieu but also hint at future trajectories and prospective avenues in the field.

**TABLE 3:** The top 10 institutions published literature related to MSC-EVs for drug delivery from 2013 to 2023.

Rank	Institution	Article counts	Percentage	Country
1	Shanghai Jiao Tong University	37	3.561	China
2	Zhejiang University	35	3.369	China
3	Harvard University	25	2.406	United States
4	University of California System	24	2.31	United States
5	Shahid Beheshti University Medical Sciences	23	2.214	Iran
6	Tehran University of Medical Sciences	22	2.117	Iran
7	Jiangsu University	21	2.021	China
8	Sichuan University	21	2.021	China
9	Central South University	20	1.925	China
10	Chinese Academy of Sciences	19	1.829	China

### 3.7 Analysis of references

**FIGURE 7:**
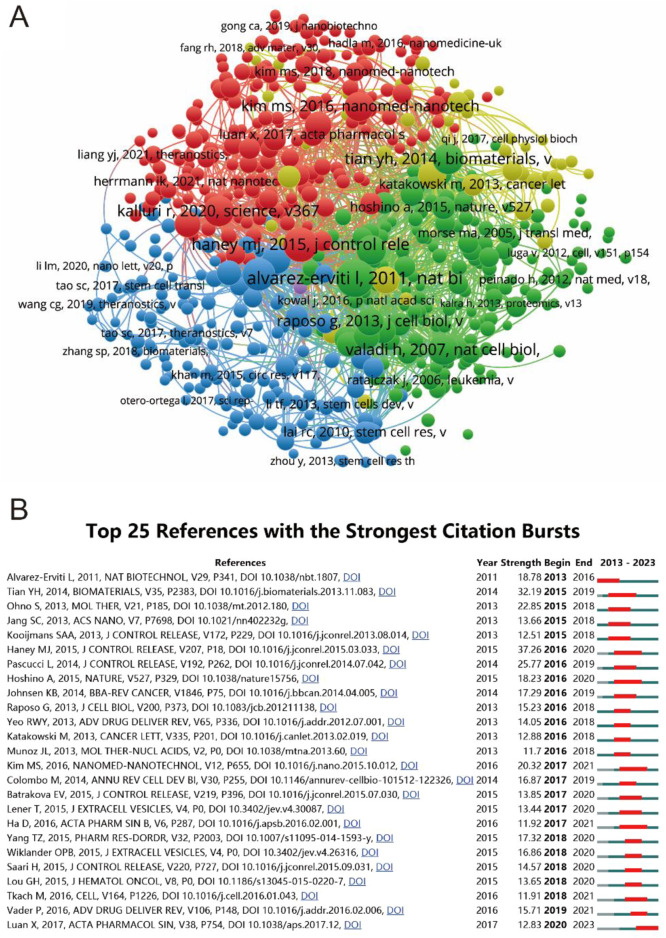
Mapping of references in studies on MSC-EVs for drug delivery spanning from 2013 to 2023. **(A)** Network visualization of reference analysis using VOSviewer. **(B)** Identification of the top 25 references exhibiting the most pronounced citation bursts in publications.

In an effort to illuminate the landscape of seminal literature within the field, a sophisticated co-citation analysis was conducted using VOSviewer, as depicted in Figure 7A. This analysis, aimed at unraveling the intricate web of scholarly interrelations based on aggregate citation metrics, scrutinized 575 references, each surpassing the threshold of being cited in over 20 documents. The quintet of articles at the apex of this evaluation, distinguished bFy their formidable total link strength, includes Alvarez-Erviti L’s 2011 publication in “Nature Biotechnology” (291 times), Haney MJ’s 2015 work in “Journal of Controlled Release” (241 times), Théry C’s 2018 article in “Journal of Extracellular Vesicles” (212 times), Tian YH’s 2014 study in “Biomaterials” (191 times), and Kalluri R’s 2020 research in “Science” (also 191 times). Complementing this, the concept of ‘citation burst’, a critical indicator highlighting references that have sparked considerable interest over specific periods, was meticulously analyzed using CiteSpace. This analysis culminated in the identification of the most potent citation bursts, with Haney MJ’s study leading the pack, exhibiting a burst strength of 37.26 from 2016 to 2020, followed by Tian YH and Pascucci L, consolidating the understanding of trends and focal points in the domain’s research trajectory as summarized in Figure 7B.

### 3.8 Co-occurrence analysis of keywords

**FIGURE 8:**
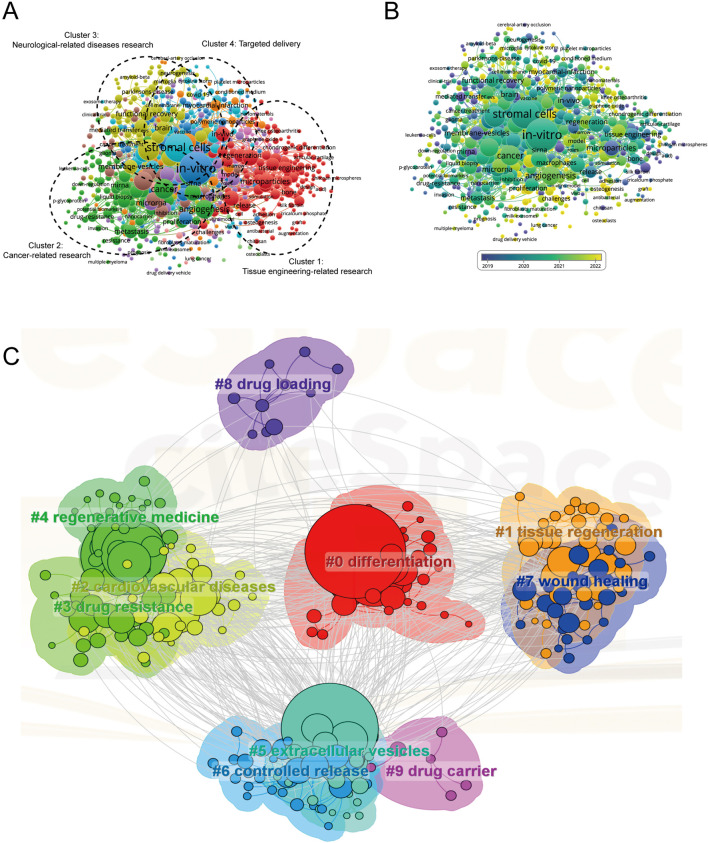
Mapping of keywords in studies on MSC-EVs for drug delivery spanning from 2013 to 2023. **(A)** Utilization of VOSviewer for network visualization of keywords, with point size representing frequency. Cluster 1 (red): research related to tissue engineering; Cluster 2 (green): cancer-related research; Cluster 3 (yellow): studies on neurological diseases; Cluster 4 (blue): research on targeted delivery. **(B)** Distribution of keywords based on mean frequency of appearance; yellow keywords appeared later than blue ones. **(C)** Visualization of keyword clustering from 2013 to 2023.

The co-occurrence analysis undertaken in this study was pivotal in elucidating the prevailing research trajectories and focal areas in scientific inquiry. This analysis, instrumental in charting the course of research evolution, leveraged keywords that appeared more than 10 times in titles and abstracts across all papers. Analyzed through VOS viewer, as depicted in Figure 8A, a total of 741 keywords were discerned and primarily categorized into four distinct clusters: Cluster 1 embodies tissue engineering-related research (red), Cluster 2 encompasses cancer-related research (green), Cluster 3 covers neurological-related diseases research (yellow), and Cluster 4 pertains to targeted delivery (blue). This categorization not only highlights the salient research themes in MSC-EVs for drug delivery but also underscores the diversity and breadth of the field.

In the tissue engineering-related cluster, prominent keywords included regenerative medicine, biomaterials, and microparticles. The cancer-related research cluster predominantly featured keywords like angiogenesis, tumor microenvironment, and metastasis. The neurological-related diseases research cluster was characterized by keywords such as mesenchymal stromal cells, functional recovery, and drug-delivery vehicles. Lastly, the targeted delivery cluster was marked by keywords like cell-derived exosomes, stromal cells, and *in-vitro*. VOS viewer further color-coded the keywords based on their average occurrence in published papers (Figure 8B), with blue signifying earlier appearances and yellow indicating more recent emergence. This color coding revealed that the trends in the four clusters have remained relatively stable, suggesting a balanced and ongoing interest across these research domains in the foreseeable future. A network map, as shown in Figure 8C, was constructed to visualize these keyword clusters, with each node representing a significant keyword. The top ten keywords, in order of prominence, were #0 differentiation, #1 tissue regeneration, #2 cardiovascular diseases, #3 drug resistance, #4 regenerative medicine, #5 extracellular vesicles, #6 controlled release, #7 wound healing, #8 drug loading, #9 drug carrier.

**FIGURE 9:**
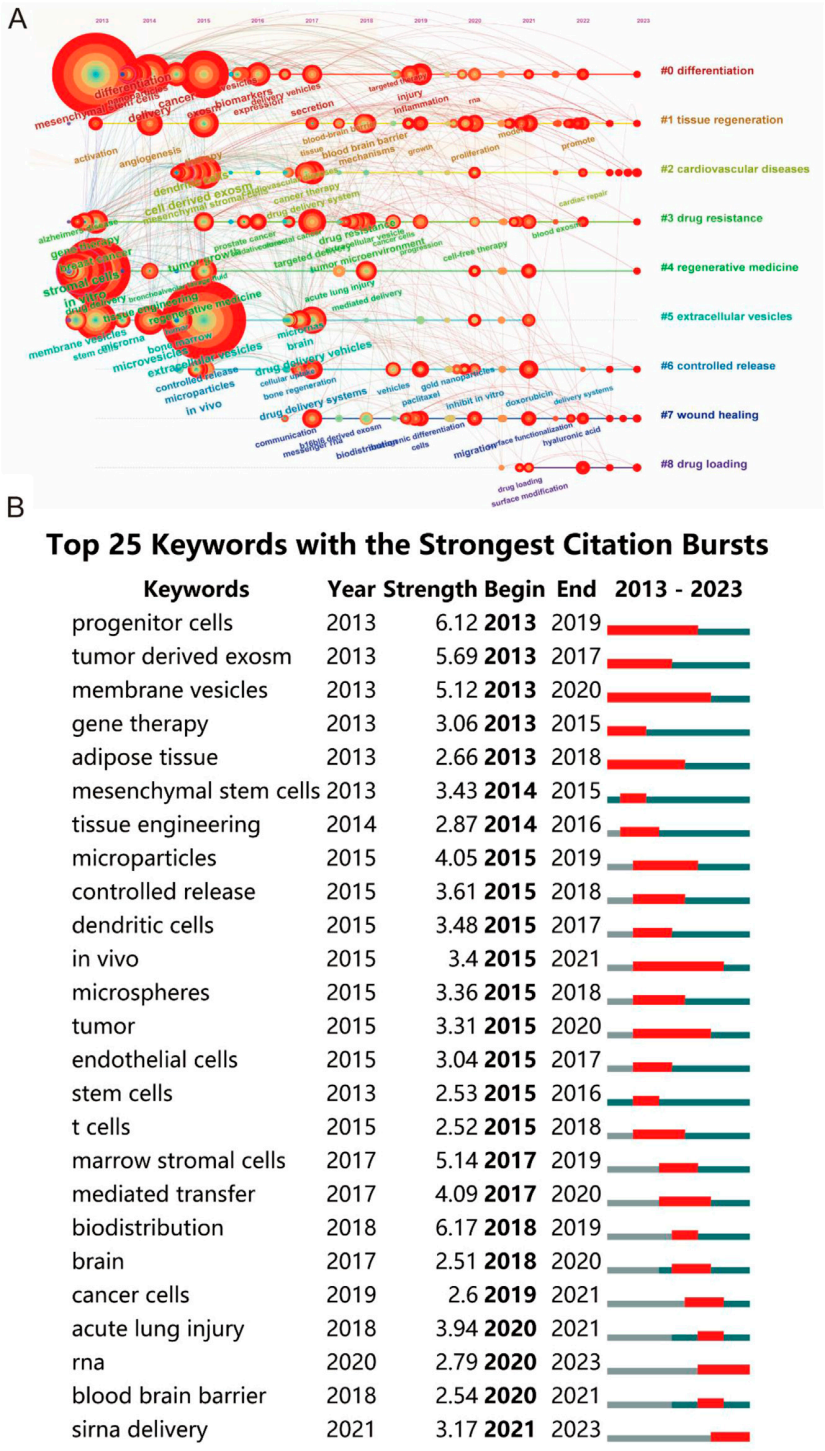
**(A)** Visualization of the timeline for keywords spanning from 2013 to 2023. **(B)** Identification of the top 25 keywords exhibiting the most pronounced citation bursts in publications.

The analysis extended to examining the transformation of keywords over the decade from 2013 to 2023, with a visualization in Figure 9A. This timeline revealed notable variations in keyword frequency over time, signifying shifts in research focus. Furthermore, using CiteSpace’s burst detection algorithm, with a minimum burst duration set at 5 years, the top 25 keywords with the strongest citation bursts were identified, as shown in Figure 9B. The blue lines in the graph represent the keyword timelines, with red segments indicating the burst duration. The keyword ‘biodistribution’ emerged as the most intense (strength = 6.17), closely followed by ‘progenitor cells’ (6.12) and ‘tumor-derived exosomes’ (5.69). Remarkably, ‘membrane vesicles’ had the longest burst duration, spanning 23 years from 1,013 to 2,020, reflecting its enduring relevance in the field.

### 3.9 Trends and hotpots of *in vivo* study on MSC-EVs delivery system

**FIGURE 10:**
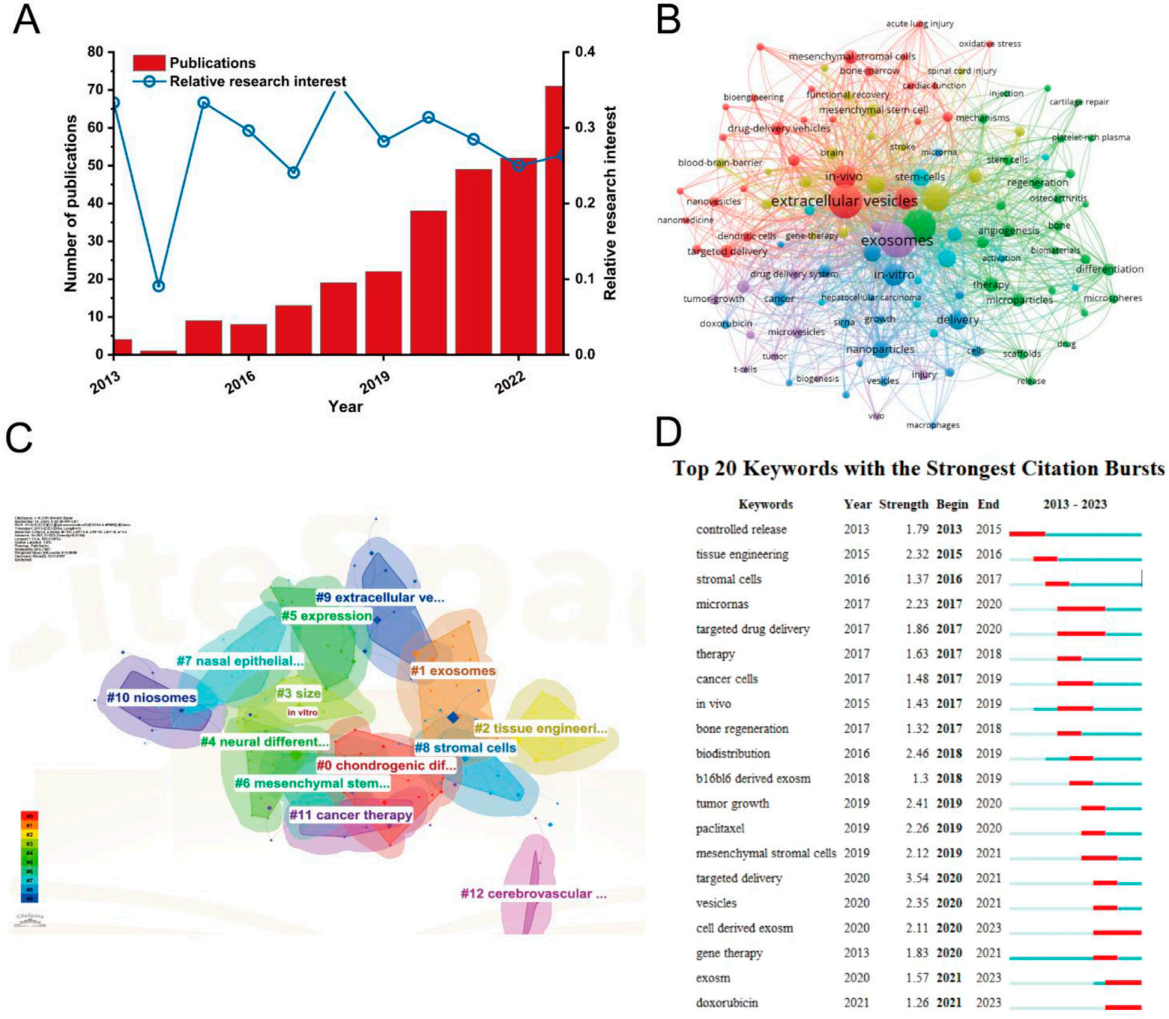
Analysis of *in vivo* studies on MSC-derived exosome delivery trends and research hotspots. **(A)** Publication count and relative research interest over time. **(B)** Co-occurrence network of keywords generated by VOSviewer. **(C)** Keyword clustering analysis using CiteSpace. **(D)** Top 20 keywords with the strongest citation bursts.

As the therapeutic applications of MSC-EVs continue to expand, understanding the key research trends and hotspots within *in vivo* studies is essential for guiding future investigations. A comprehensive analysis of the literature reveals critical areas of focus that are shaping the development of MSC-EV delivery systems for clinical use. In Figure 10A, the publication count and relative research interest indicate a significant upward trend in the number of studies on *in vivo* delivery of MSC-derived exosomes, particularly in the past 5 years. This rise reflects the growing recognition of MSC-derived exosomes as promising candidates for drug delivery and regenerative therapies. The corresponding increase in RRI further emphasizes the burgeoning interest within the scientific community. Figure 10B presents a co-occurrence network of keywords generated by VOSviewer. The network reveals key themes such as “exosome delivery,” “regenerative medicine,” “tumor microenvironment,” and “drug delivery systems.” These themes suggest that current research is heavily focused on leveraging MSC-derived exosomes for tissue repair, cancer treatment, and targeted therapeutic delivery. In Figure 10C, the CiteSpace clustering analysis identifies distinct research areas, including stem cell therapy, targeted delivery, and cancer therapy. This analysis illustrates the breadth of applications explored within MSC-derived exosome *in vivo* studies, ranging from regenerative medicine to oncology. Figure 10D highlights the top 20 keywords with the strongest citation bursts, indicating specific topics that have gained significant attention in recent years. Keywords like “tumor-derived exosomes,” “angiogenesis,” and “nanoparticle delivery” are among the strongest citation bursts, signaling the importance of targeted exosome delivery systems and their role in modifying the tumor microenvironment.

## 4 Discussions

### 4.1 Trend of global publications

MSCs have garnered significant attention in both basic and clinical research due to their potential in treating various diseases, as highlighted by Blau and Daley (Blau and Daley, 2019). However, challenges such as the complexities of long-term *ex vivo* culture, increased immunogenicity in differentiated cells, and limited therapeutic efficacy have been identified by Musiał-Wysocka et al. (Musiał-Wysocka et al., 2019), constraining their broader application. These limitations have catalyzed the exploration of alternative, cell-free therapies. Among these, EVs have emerged as a promising substitute. EVs, including diverse forms like exosomes, microvesicles, and apoptotic bodies, are cell-derived nanoparticles capable of mirroring certain cellular functions. Their advantages over traditional MSC therapies include comparable therapeutic effects, reduced immunogenicity, enhanced safety, minimal side effects, effective protection of therapeutic agents, simpler preservation, fewer ethical concerns, and the ability to traverse biological barriers, as detailed by Nguyen et al. (2021).

The field of EVs, particularly in the context of MSCs, has witnessed a surge in research activity. This is exemplified by the comprehensive reviews and studies conducted by various researchers. Wang et al. (2020) provided an insightful overview of global trends and developments in the exosome domain, a key component of EVs, from 1994 to 2017. Furthermore, Zhang X. et al. (2022) delved into MSC-EVs, uncovering a steep growth trend in research focused on their therapeutic effects and mechanisms. Their work also forecasts the future direction of this field, anticipating an increase in multidisciplinary integration and emphasis on senescence-related topics. MSCs, known for their multi-directional differentiation and self-renewal capabilities, are a subset of adult stem cells originating from the mesoderm (Li and Hua, 2017). Yet, a comprehensive global analysis and visualization of EVs derived not just from MSCs, but from a broader spectrum of stem cells, remains an area less explored.

To bridge this gap, our study employed bibliometric and visualized analysis techniques to encapsulate the current status and project future trends in the realm of stem cell-based EV research. Our findings reveal a marked increase in the volume of publications per year in this domain, alongside a significant rise in Research Relative Index (RRI) values, indicating a growing interest and investment in this field. Our analysis also showed that this research is a global endeavor, with contributions from approximately 73 countries and 1,479 institutions. Notably, China and the United States are leading this research front, collectively accounting for over half of the publications. Such a concentrated effort in MSC-derived EV research underscores its status as a burgeoning field of study. Given the increasing evidence of the paracrine effects of mesenchymal stem cells mediated through EV secretion (Baglio et al., 2012) and their observed intercellular communication capabilities under various conditions (Chang et al., 2018), it is reasonable to anticipate a continued escalation in detailed studies on stem cell-based EVs, guiding researchers towards high-caliber, innovative investigations.

### 4.2 Quality and status of global publications

To elucidate the academic impact and quality of research contributions from various countries in the field of EVs derived from MSCs, an in-depth analysis was conducted focusing on total citations and H-index, with results showcased in Figure 3. Despite China leading in publication volume with 445 papers, the United States, with 207 publications, boasts the highest total citation count of 16,800. The H-index comparison reveals a marginal disparity between China and the United States, signifying that both nations maintain a prominent research profile, with the United States garnering more extensive global attention. Intriguingly, England excels in average citations, surpassing the United States and Australia, suggesting the pivotal role of the United States in this specialized domain. Moreover, China’s ascendancy in both publication count and H-index, a significant leap from its fifth rank in 2019 (Zhang H. et al., 2022), underscores its growing global recognition in this research area. This balance of quantity and quality in Chinese publications may be attributed to recent shifts in the Chinese Academic Evaluation Systems (CAESs) towards a dual focus (Synnestvedt et al., 2005). Nevertheless, China still faces challenges in elevating the quality of its research output, a goal potentially achievable through CAES reforms and increased research funding (Xing et al., 2018).

An investigation into the journals associated with these publications, as depicted in Figure 6, reveals Pharmaceutics, Journal of Controlled Release, International Journal of Molecular Sciences, Frontiers in Bioengineering and Biotechnology, and International Journal of Nanomedicine as the leading publishers in this sphere. The close publication counts among these journals suggest they could be key venues for future research (Table 3). Furthermore, the significant contributions of leading institutes from the top 10 countries, particularly those in China and the United States, highlight the crucial role of premier research institutions in enhancing a country’s academic stature. The predominance of Chinese authors and institutions, notably Shanghai Jiao Tong University (Table 4), coupled with substantial funding from the National Natural Science Foundation of China (NSFC) (Table 1), emphasizes China’s pivotal role in this research area. These authors, listed in Table 5, could be focal points for accessing the latest advancements in the field.

**TABLE 4:** The top 10 funds related to MSC-EVs for drug delivery from 2013 to 2023.

Rank	Journal	Article counts	Percentage
1	National Natural Science Foundation of China NSFC	283	27.238
2	United States Department of Health Human Services	84	8.085
3	National Institutes of Health NIH United States of America	83	7.988
4	European Union Eu	30	2.887
5	National Research Foundation of Korea	25	2.406
6	National Key Research and Development Program of China	23	2.214
7	China Postdoctoral Science Foundation	20	1.925
8	Fundamental Research Funds for The Central Universities	19	1.829
9	Spanish Government	18	1.732
10	United Kingdom Research Innovation UKRI	15	1.444

**TABLE 5:** The top 10 most productive journals related to MSC-EVs for drug delivery from 2013 to 2023.

Rank	Journal	Article counts	Percentage	Impact factor (IF, 2023)
1	Pharmaceutics	37	3.561	5.4
2	Journal of Controlled Release	35	3.369	10.8
3	International Journal of Molecular Sciences	34	3.272	5.6
4	Frontiers in Bioengineering and Biotechnology	27	2.599	5.7
5	International Journal of Nanomedicine	19	1.829	8.0
6	Advanced Drug Delivery Reviews	18	1.732	16.1
7	Cells	17	1.636	6.0
8	Journal of Nanobiotechnology	16	1.54	10.2
9	Stem Cell Research Therapy	16	1.54	7.5
10	Acta Biomaterialia	14	1.347	9.7

Employing bibliometric research methods, this study establishes similarity relationships among publications across various parameters including institution, journal, country, and author. Figures 4–6 demonstrate China’s prominent position, with Shanghai Jiao Tong University and author Zhang Y being highly influential. The International Journal of Molecular Sciences emerges as a key journal in this domain. Additionally, co-citation analysis, examining publication impacts through total citation counts, reveals a high research frequency in pharmacology studies related to MSC-EVs, with the journal BLOOD potentially leading in citation frequency. Co-author analysis, aimed at identifying collaborative networks, indicates that authors, institutions, and countries with higher total link strength are more inclined towards collaboration. Based on these findings, the study offers insights and recommendations for future research, highlighting the active involvement of institutions like Shanghai Jiao Tong University and Zhejiang University from China, and Harvard University from the United States. The robust connections between these entities, primarily in China and the United States, may significantly influence research direction and quality enhancement in this burgeoning field.

### 4.3 Keyword trends and their relevance to MSC-EV drug delivery

In the burgeoning field of MSC-EVs, co-occurrence analysis has unveiled key research directions and prevailing themes. This analysis, grounded in the examination of keywords within the titles and abstracts of pertinent studies, culminated in the construction of a network map of occurrences. As delineated in Figure 8A, four predominant research trends emerged: tissue engineering, cancer research, neurological diseases, and targeted delivery. These trends not only resonate with the established understanding within the field but also offer clarity on potential avenues for future research endeavors.

In the tissue engineering cluster, MSC-EVs have emerged as powerful tools in regenerative medicine. By promoting tissue repair and regeneration, MSC-EVs have found applications in the development of tissue-engineered constructs. Keywords such as regenerative medicine, biomaterials, and microparticles underscore the diversity of approaches used to harness their therapeutic potential. For example, the integration of MSC-EVs with biomaterials has enhanced the delivery of these vesicles to damaged tissues, improving the effectiveness of cell-based therapies. Moreover, microparticles have provided innovative delivery mechanisms, allowing for the controlled release of MSC-EVs, thus prolonging their therapeutic effects. Such developments are especially relevant for regenerative applications in cartilage, bone, and skin, where the ability to accelerate tissue repair remains a priority in clinical settings.

The cancer research cluster highlights the dual role of MSC-EVs as both therapeutic agents and diagnostic biomarkers in oncology. Keywords such as angiogenesis, tumor microenvironment, and metastasis reflect the multifaceted nature of MSC-EV applications in cancer treatment. MSC-EVs have demonstrated their capacity to selectively target tumors and influence the tumor microenvironment, either inhibiting or promoting angiogenesis depending on the therapeutic context. Their ability to modulate the immune response and suppress tumor growth further solidifies their potential in oncology. Importantly, MSC-EVs are being explored for their role in preventing metastasis, by delivering anti-cancer agents directly to tumor cells, thus reducing the likelihood of secondary tumor formation. The capacity of MSC-EVs to carry tumor-specific proteins and nucleic acids also makes them promising candidates for early cancer detection and personalized therapies.

In the neurological diseases cluster, MSC-EVs are being explored as potential therapies for a range of central nervous system disorders, including neurodegenerative diseases, stroke, and traumatic brain injury. Keywords like mesenchymal stromal cells, functional recovery, and drug-delivery vehicles emphasize the regenerative potential of MSC-EVs in neural repair. Their ability to cross the blood-brain barrier—a significant challenge in neurological treatment—enhances their value in promoting functional recovery by reducing inflammation, stimulating neurogenesis, and improving the survival of neural cells. Studies have shown promising results in animal models, suggesting MSC-EVs could play a key role in developing therapies for stroke and neurodegenerative conditions. Furthermore, MSC-EVs are being engineered to deliver neuroprotective drugs and genes, potentially improving the precision and efficacy of treatments aimed at mitigating the effects of neurodegeneration.

Finally, the targeted delivery cluster underscores the innovations in MSC-EV-based delivery systems, where keywords such as cell-derived exosomes, stromal cells, and *in-vitro* reflect the ongoing efforts to enhance the specificity and efficiency of therapeutic delivery. MSC-EVs, particularly exosomes, have been widely recognized for their inherent targeting capabilities, driven by the surface proteins that allow them to home in on specific cell types. The ability to engineer these vesicles to carry bioactive molecules, including proteins and RNAs, positions them as ideal candidates for delivering therapeutic agents to precise locations in the body. Preclinical *in-vitro* studies have been instrumental in refining these delivery systems, providing insights into their uptake and therapeutic efficacy before transitioning to *in-vivo* and clinical trials. These advancements are critical in ensuring the safety and effectiveness of MSC-EV-based therapies for various conditions.

The centrality of specific keywords within the co-occurrence map underscores the need for advanced, high-quality research within these identified areas. Complementing this, an overlay visualization map, akin to the co-occurrence map but distinct in its color-coded representation, was developed, as shown in Figure 8C. The varied hues signify different scores, hinting at the likelihood of these themes evolving into focal topics of future research. Recent scholarly activities have seen a surge in studies involving various forms of EVs and mesenchymal stem cells, such as exosomes, microvesicles, and ectosomes, highlighting their increasing relevance (Shao et al., 2018; Lin et al., 2021; Phinney et al., 2015). Keyword burst detection further refined this analysis, identifying ‘progenitor cells’, ‘tumor-derived exosomes’, and ‘membrane vesicles’ as areas of intense research interest, as evident in Figures 9A, B.

### 4.4 Research frontiers and hotspots

The top 10 research and review articles with the most citations have been listed in Tables 6, 7. It shows that EVs have consistently been at the forefront of biomedical research, celebrated for their pivotal roles in a wide array of diseases. These nanoscale carriers, loaded with diverse biomolecules and encapsulated by phospholipid bilayers, are integral to the field of precision medicine, functioning as natural vehicles for the delivery of pharmaceutical agents (Wang et al., 2017). Recognized as a heterogeneous group of lipid-bound nanoparticles, EVs are involved in various (patho) physiological processes and are increasingly being explored for their potential to deliver therapeutic agents to specific cells or tissues. Their therapeutic applications are broad and include the alleviation of osteoarthritis (Chen L. et al., 2019; Khan et al., 2015; Tavakoli Dargani and Singla, 2019), mediation of cartilage repair, enhancement of cardiac function following myocardial infarction (Chen W. et al., 2019; Gao et al., 2020), and promotion of healing in pressure ulcers (Zhang et al., 2019; Luo et al., 2023; Tang et al., 2022).

**TABLE 6:** The top 10 research articles with the most citations in the field of MSC-EVs for drug delivery from 2013 to 2023.

Rank	Title	Journal	If	Publication year	Total citations	Average citation per year
1	Extracellular vesicles: biology and emerging therapeutic opportunities	Nature Reviews Drug Discovery	120.1	2013	2,308	230.80
2	1.1.1 Using exosomes, naturally-equipped nanocarriers, for drug delivery	Journal of Controlled Release	10.8	2015	662	82.8
3	Surface functionalized exosomes as targeted drug delivery vehicles for cerebral ischemia therapy	Biomaterials	14.0	2018	659	131.8
4	Paclitaxel is incorporated by mesenchymal stromal cells and released in exosomes that inhibit *in vitro* tumor growth: A new approach for drug delivery	Journal of Controlled Release	10.8	2014	620	68.9
5	High-resolution proteomic and lipidomic analysis of exosomes and microvesicles from different cell sources	Journal of Extracellular Vesicles	16.0	2016	445	63.6
6	Delivery of Functional Anti-miR-9 by Mesenchymal Stem Cell-derived Exosomes to Glioblastoma Multiforme Cells Conferred Chemosensitivity	Molecular Therapy-Nucleic Acids	8.8	2013	399	39.9
7	Exosome Mediated Delivery of miR-124 Promotes Neurogenesis after Ischemia	Molecular Therapy-Nucleic Acids	8.8	2017	381	63.5
8	Mesenchymal Stromal Cell Exosomes Ameliorate Experimental Bronchopulmonary Dysplasia and Restore Lung Function through Macrophage Immunomodulation	American Journal of Respiratory and Critical Care Medicine	24.7	2018	380	76.0
9	Exosome-Liposome Hybrid Nanoparticles Deliver CRISPR/Cas9 System in MSCs	Advanced Science	15.1	2018	338	67.6
10	Elucidation of Exosome Migration Across the Blood-Brain Barrier Model *In Vitro*	Cellular and Molecular Bioengineering	2.8	2019	316	79.0

**TABLE 7:** The top 10 review articles with the most citations in the field of MSC-EVs for drug delivery from 2013 to 2023.

Rank	Title	Journal	If (2023)	Publication year	Total citations	Average citation per year
1	Exosomes in cancer development, metastasis, and drug resistance: a comprehensive review	Cancer and Metastasis Reviews	9.2	2013	842	84.2
2	Exosomes: Therapy delivery tools and biomarkers of diseases	Pharmacology and Therapeutics	13.5	2017	691	115.2
3	Mesenchymal stem cell: An efficient mass producer of exosomes for drug delivery	Advanced Drug Delivery Reviews	16.1	2013	585	58.5
4	Hydrogel microparticles for biomedical applications	Nature Reviews Materials	83.5	2020	555	185.0
5	Degradability and Clearance of Silicon, Organosilica, Silsesquioxane, Silica Mixed Oxide, and Mesoporous Silica Nanoparticles	Advanced Materials	29.4	2017	533	88.8
6	Advances in therapeutic applications of extracellular vesicles	Science Translational Medicine	17.1	2019	529	132.3
7	Exosomes: Vehicles of Intercellular Signaling, Biomarkers, and Vectors of Cell Therapy	Annual Review of Physiology	18.2	2015	504	63.0
8	Exosome: A Review of Its Classification, Isolation Techniques, Storage, Diagnostic and Targeted Therapy Applications	International Journal of Nanomedicine	8.0	2020	501	167.0
9	Extracellular vesicles as drug delivery systems: Why and how?	Advanced Drug Delivery Reviews	16.1	2020	498	166.0
10	Promoting tissue regeneration by modulating the immune system	Acta Biomaterialia	9.7	2017	440	73.3

The intrinsic potential of EVs as natural delivery vehicles has spurred the development of bioengineering techniques aimed at maximizing their therapeutic efficacy (Evers et al., 2022). This enhancement can be achieved through two primary strategies: cargo engineering and surface engineering. By customizing the therapeutic payload of EVs or enhancing their selectivity for target cells, bioengineered EVs have the potential to become highly personalized and targeted therapeutic agents. Current research explores EVs as natural nanocarriers through artificial loading with a variety of therapeutic agents, including small molecules, drugs, proteins, and different RNA species such as small interfering RNA (siRNA) and microRNA (miRNA) (Table 8). Incorporating extrinsic cargo into EVs involves manipulating either the EVs themselves or the parental cells from which they originate. This can be done using two main methods: exogenous (direct) loading and endogenous (indirect) loading. Exogenous loading occurs after EV isolation and involves directly encapsulating the desired therapeutic cargo through processes such as co-incubation (Didiot et al., 2016; Sun et al., 2010), electroporation, sonication, freeze-thawing, extrusion, and permeation using detergent-based compounds (Didiot et al., 2016; Goh et al., 2017; Shtam et al., 2013; Haney et al., 2015). Endogenous loading, on the other hand, relies on the presence of the desired cargo within the producer cell, which then uses its cellular machinery to incorporate the cargo into the EVs naturally during their biogenesis.

**TABLE 8:** MSC-EVs for common drugs delivery.

Drugs		Loading methods	Reference
Small Molecule Drugs	paclitaxel, doxorubicin	Drug Priming, Electroporation, Co-incubation	Pascucci et al. (2014), Abas et al. (2022), Banche Niclot et al. (2023), Yang et al. (2022b), Wang et al. (2022), Bagheri et al. (2020)
Nucleic Acid Drugs	siRNA, miRNA, mRNA	Electroporation, Liposome Transfection, Co-incubation	Hinkelmann et al. (2022), Deng and Ke (2023), Liu et al. (2022), Naseri et al. (2018), Sun et al. (2023), Liu et al. (2023), Altanerova et al. (2019)
Protein and Peptide Drugs	Growth factors, Perforin, Antibodies	Chemical Conjugation, Genetic Engineering, Co-incubation	Lopatina et al. (2014), Schultz et al. (2015)
Natural Compounds and Metabolites	Anti-inflammatory drugs (corticosteroids), Antioxidants	Co-incubation, Chemical Modification	Li et al. (2021), Dashnyam et al. (2018), Luo et al. (2021), Li et al. (2024), Jiyah et al. (2024)
CRISPR/Cas9 Gene Editing System	Cas9 nucleases, gRNA	Electroporation, Liposome Transfection, Genetic Engineering	Hazrati et al. (2022), Dubey et al. (2024), Ye et al. (2020)

However, EVs administered *in vivo* face the challenge of rapid clearance, primarily through uptake by cells in organs such as the liver, spleen, gastrointestinal tract, and lungs (Wiklander et al., 2015). The surface characteristics of EVs are crucial for their biodistribution, tissue tropism, and therapeutic efficacy. Modifying the EV surface can enhance their targeting to specific cell types, enable them to traverse various biological barriers, and extend their lifespan *in vivo* until they reach the target location (Jia et al., 2018; Kamerkar et al., 2017). Several strategies have been investigated to functionalize the surface of EVs, including genetic manipulation (engineering parental cells to produce EVs with transmembrane targeting moieties), chemical modification (anchoring targeting moieties to the surface of isolated EVs), and hybrid membrane engineering (conjugating natural EVs with synthetic liposome nanoparticles). Despite these advances, most methods for bioengineering the content and surface of MSC-derived EVs remain at the pre-clinical stage and face scalability challenges. Several obstacles hinder their therapeutic application, including inefficient production at clinical grade, lack of standardized methods for isolation, characterization, and quantification, inaccurate cargo characterization, pharmacokinetics issues, insufficient targeted delivery, limited drug loading efficiencies, EV heterogeneity, and safety profiles (Gowen et al., 2020; Meng et al., 2020). To facilitate the clinical translation of bioengineered MSC-EVs, the simple, cost-effective, and streamlined manufacturing process is urgently needed in the future (de Almeida Fuzeta et al., 2022; Herrmann et al., 2021).

The analysis of *in vivo* studies on MSC-derived exosome delivery (Figure 10) underscores several key research trends and hotspots. The sustained increase in publication volume and RRI reflects a growing interest in the clinical potential of MSC-derived exosomes. Keyword co-occurrence and clustering analysis reveal that much of the research is focused on therapeutic applications, particularly in regenerative medicine and cancer therapy. This is reinforced by the strong citation bursts for topics related to targeted delivery and the modulation of the tumor microenvironment, highlighting the potential of MSC-derived exosomes as both therapeutic agents and drug delivery vehicles. As the field continues to expand, *in vivo* studies will likely concentrate on optimizing the delivery efficiency and therapeutic efficacy of MSC-derived exosomes, particularly for cancer treatment and regenerative applications. The increasing focus on specific delivery mechanisms, such as nanoparticle-based systems and the targeting of tumor-derived exosomes, suggests that the next phase of research will aim to overcome current challenges related to bioavailability and precise targeting of therapeutic exosomes *in vivo*.

### 4.5 Limitations of this study

The current paper, while offering significant insights into the global research trends and interests in MSC-EVs for drug delivery, has limitations that merit careful consideration. A primary challenge lies in extracting detailed information about MSC-EVs, specifically their origins and intricate interrelationships within the context of drug delivery. This complexity poses a barrier to fully understanding the nuances and potential applications of MSC-EVs in this field. Furthermore, there is a potential risk of omitting relevant publications due to the constraints of database and language selection. The exclusion of major databases such as PubMed, Embase, Cochrane, and non-English language databases from the study might result in a skewed representation of the research landscape. This limitation could lead to an inadvertent oversight of pivotal studies and findings that are not indexed in the included databases or are published in languages other than English. Additionally, the reliance on citation counts as a measure of a paper’s impact can be problematic. Newly published papers, despite potentially being of high quality, may not have accumulated enough citations to be recognized in bibliometric analyses. This gap might create a disparity between the actual ongoing research activities and the trends observed through bibliometric methods. To address these limitations, it is crucial to broaden the scope of future research in this domain. This expansion would include incorporating the latest publications, regardless of their current citation count, and ensuring that non-English language papers are also considered. Such an inclusive approach would provide a more comprehensive and accurate reflection of the global research efforts and advancements in the field of MSC-EVs for drug delivery.

## 5 Conclusion

In conclusion, our comprehensive bibliometric and visualized study underscores the burgeoning interest and remarkable advancements in the field of MSC-EVs for drug delivery from 2013 to 2023. The analysis, spanning over a decade, reveals an ascending trajectory in global research efforts, with China and the United States leading in various key metrics, including publication count, H-index, total citations, and funding. The study identifies four major research clusters - tissue engineering, cancer, neurological diseases, and targeted delivery, further delineated into ten thematic areas such as differentiation, tissue regeneration, and drug resistance. These findings not only reflect the dynamic and evolving nature of MSC-EV research but also highlight its growing prominence in the scientific community. This study provides invaluable insights into the current state and potential future directions of MSC-EV research in drug delivery, offering a roadmap for researchers and policymakers to navigate this rapidly expanding field. As MSC-EVs continue to gain traction in therapeutic applications, their role in revolutionizing drug delivery systems becomes increasingly evident, paving the way for novel and more effective treatment modalities.

## Data Availability

The original contributions presented in the study are included in the article/supplementary material, further inquiries can be directed to the corresponding authors.
